# Endoscopic Ultrasound-guided oesophago-gastric anastomosis for management of complete lumen occlusion post-oesophagectomy

**DOI:** 10.1055/a-2885-8030

**Published:** 2026-06-17

**Authors:** Giuseppe Dell'Anna, Francesca Bernardi, Gabriele Altieri, Paolo Biamonte, Francesco Vito Mandarino, Silvio Danese, Gianfranco Donatelli

**Affiliations:** 1 Gastroenterology and Gastrointestinal Endoscopy UnitIRCCS San Raffaele InstituteMilanItaly; 2 Faculty of Medicine and Surgery 18985 Vita-Salute San Raffaele UniversityMilanItaly; 3 Gastroenterology and Gastrointestinal Endoscopy Unit 476648 IRCCS Policlinico San DanatoSan Donato MilaneseItaly; 4 Interventional Endoscopy Unit 55727 Ramsay Santé Private Hospital of PeupliersParisFrance; 5 Gastroenterology and Gastrointestinal Endoscopy UnitIRCCS San Raffaele InstituteMilanoItaly; 6 Department of Clinical Medicine and SurgeryUniversity of Naples "Federico II"NaplesItaly

Complete anastomotic stenosis (AS) after esophagectomy represents a challenging condition in which conventional endoscopic treatments are often not feasible, and surgical revision carries substantial morbidity 1​ ​2​ . This video aims to demonstrate the role of endoscopic ultrasound (EUS)-guided esophagogastric anastomosis as a minimally invasive therapeutic strategy, enabling lumen restoration and facilitating the management of distally migrated esophageal stents 3​ .

We report the management of a case of complete esophagogastric AS with distal migration of two fully covered self-expandable metal stents (FC-SEMSs), managed with an EUS-guided esophagogastric anastomosis ( Video 1 ).

**Video 1** Endoscopic ultrasound-guided oesophagogastric anastomosis for the management of complete lumen occlusion post-oesophagectomy.


**Fig. 1:**
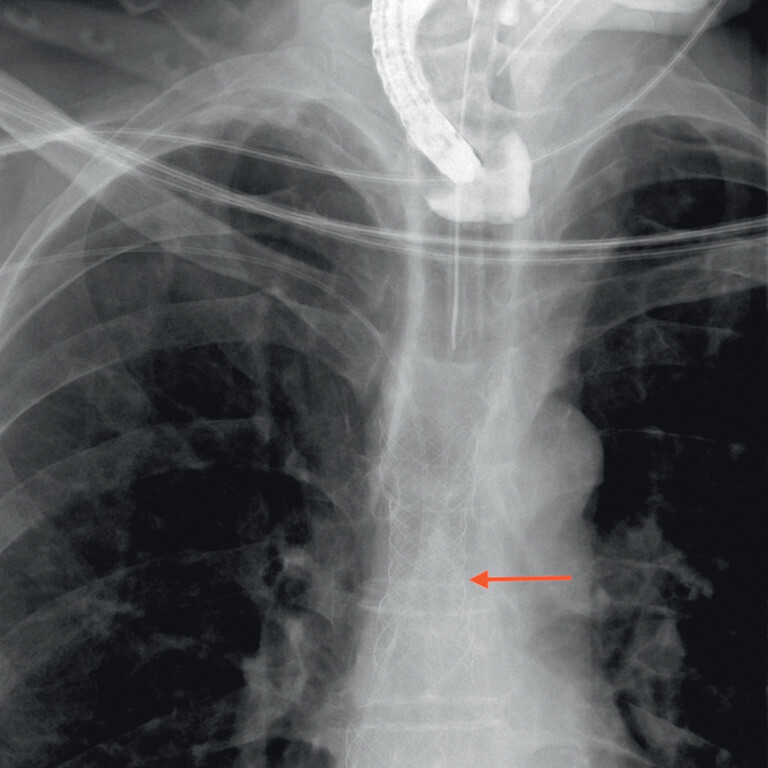
A fluoroscopic view of esophageal fully covered-self expanding metal stents (red arrow) migrated into the gastric conduit and complete stenosis of the anastomosis.

A 65-year-old woman who had undergone a three-stage esophagectomy with cervical anastomosis for squamous cell carcinoma developed a severe AS 6 months postoperatively. After unsuccessful pneumatic dilation for refractory AS, she underwent FC-SEMS placement with a stent-in-stent technique without any anti-migration system at another center 4​ . She was referred to our unit for complete AS and distal migration of both FC-SEMSs into the gastric conduit ( **Figs.** 1 **,** 2 ). After a multidisciplinary discussion, the patient was deemed a candidate for an EUS-guided de novo esophagogastric anastomosis creation 5​ .

**Fig. 2:**
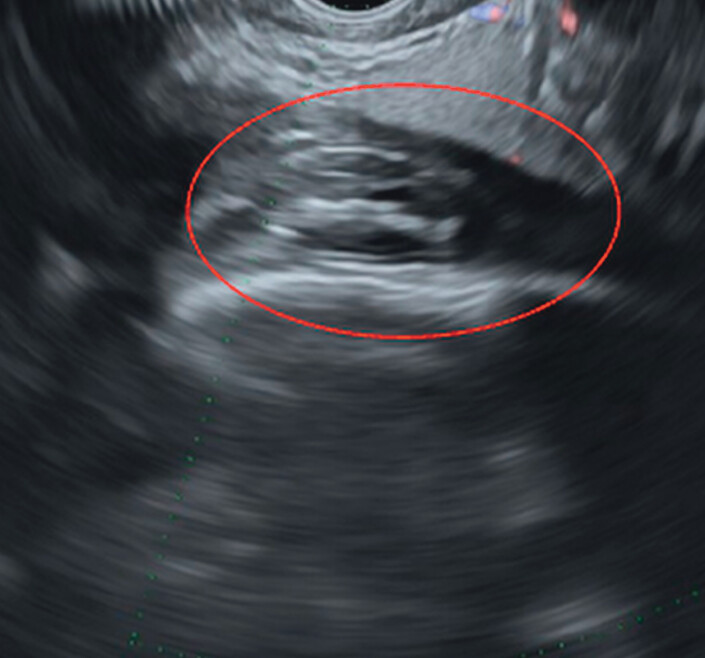
An endoscopic ultrasound view of the gastric conduit lumen (red circle) distal to the complete anastomotic stenosis.

**Fig. 3:**
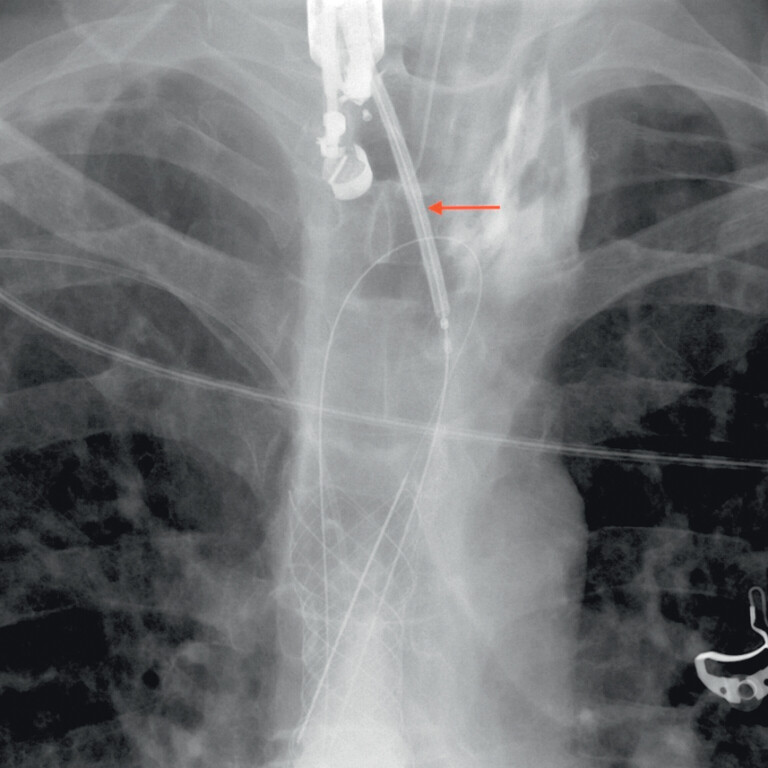
A fluoroscopic view of 10Fr cystotome (red arrow) advanced through the stenosis to the gastric conduit lumen to create a stable esophagogastric tract.

The creation of a de novo esophagogastric anastomosis was performed under EUS guidance using a 19-G needle to access the gastric conduit lumen, followed by guidewire advancement, and the 10Fr cystotome-assisted creation of a neoesophagogastric tract ( Fig. 3 ).

**Fig. 4:**
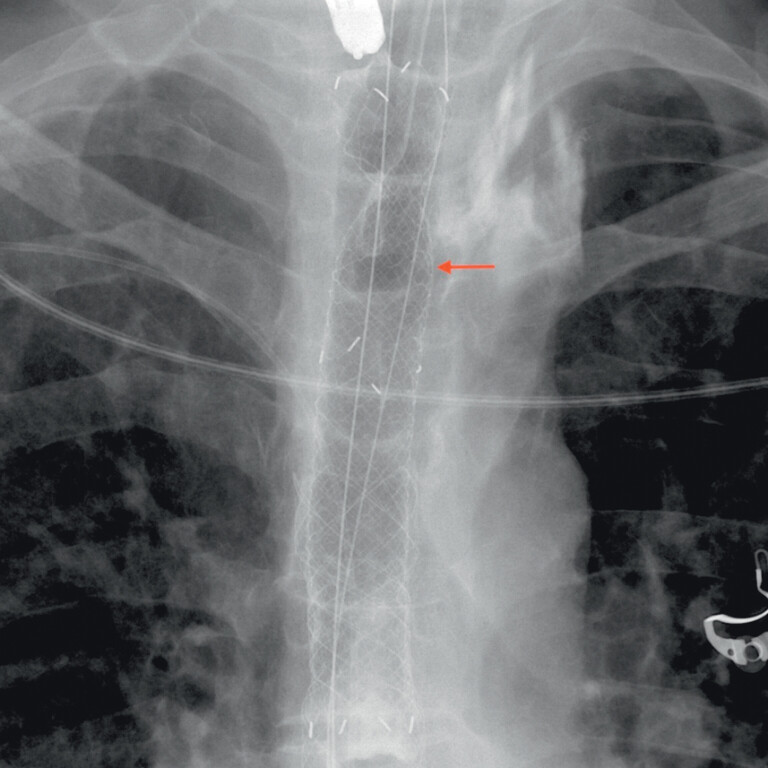
A fluoroscopic view of the new esophageal 20 mm×11 cm fully covered-self expanding metal stent (red arrow) placement after restoring luminal patency.

A pneumatic dilatation of the esophagogastric tract was performed, and the previously migrated FC-SEMSs were removed. A new FC-SEMS was deployed across the neoanastomosis without fixation, given the expected short indwelling time and to avoid additional procedural complexity, to restore and maintain luminal patency ( Fig. 4 ). No immediate or delayed adverse events were observed. At 1-month follow-up endoscopy, the newly created anastomotic tract was patent. Although the FCSEMS had partially migrated into the gastric lumen, stent removal was straightforward, and the tract subsequently underwent pneumatic dilation of up to 18 mm. After 6 months of follow-up, no symptom recurrence was recorded.

**Fig. 5:**
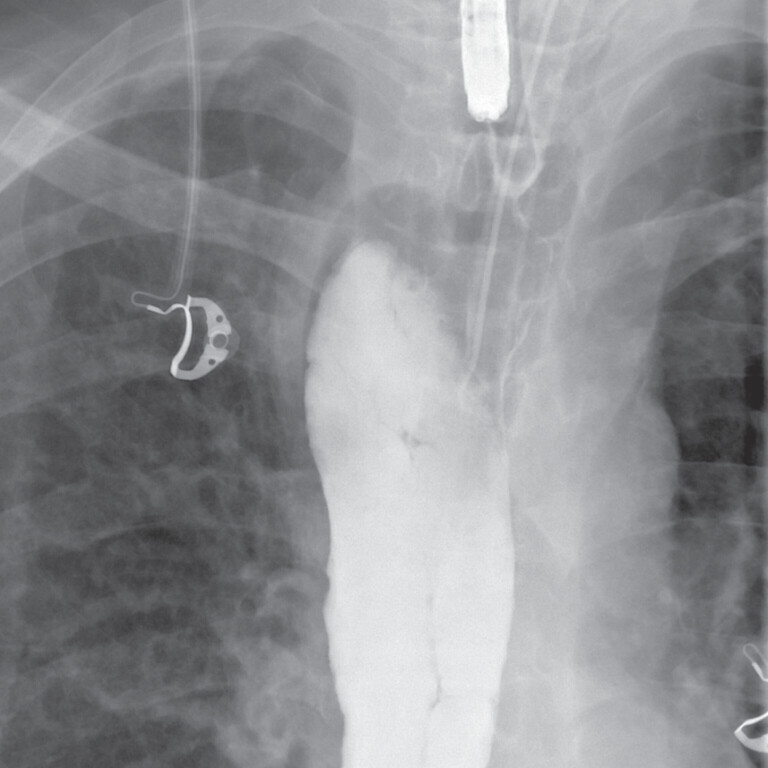
A fluoroscopic view of the final treatment outcome after 1 month.

EUS-guided esophagogastric anastomosis as a rescue strategy proved to be a feasible, safe, and effective approach for managing complete anastomotic obstruction with distal stent migration in a setting where standard endoscopic access was impossible. The favourable 6-month follow-up further supports the reliability and durability of this technique ( Fig. 5 ). This technique represents a valuable minimally invasive alternative to high-morbidity surgical revision and broadens therapeutic options for complex post-esophagectomy strictures.

Endoscopy_UCTN_Code_TTT_1AS_2AK
